# Relationship Between Prohormone Brain Natriuretic Peptide (NT-proBNP) Level and Severity of Pulmonary Dysfunction in Patients With Chronic Congestive Heart Failure

**DOI:** 10.15171/jcvtr.2015.05

**Published:** 2015-03-29

**Authors:** Masoud Nazemiyeh, Akbar Sharifi, Farhad Amiran, Leili Pourafkari, Mohammadreza Taban Sadeghi, Hossein Namdar, Mohsen Abbasnezhad

**Affiliations:** ^1^ Tuberculosis and Lung Research Center, Tabriz University of Medical Sciences, Tabriz, Iran; ^2^ Department of Internal Medicine, Tabriz University of Medical Sciences, Tabriz, Iran; ^3^ Cardiovascular Research Center, Tabriz University of Medical Sciences, Tabriz, Iran

**Keywords:** Congestive Heart Failure, NT-proBNP, Respiratory Function Tests, Oscillometry

## Abstract

***Introduction:*** Congestive heart failure (CHF) is a common disease and its prevalence is increasing in industrialized countries. NT-proBNP measurement is an established diagnostic test for diagnosis of CHF in patients who present to emergency room with acute dyspnea. The primary object of this study was to determine the relationship between levels of brain natriuretic peptide precursor and severity of lung function impairment in patients with chronic CHF.

***Methods:*** This cross-sectional and analytical study that performed in Tuberculosis and Lung Disease Research Center of Tabriz University of Medical Sciences on 95 patients with chronic heart failure, and relation between NT-proBNP levels and pulmonary function parameters were examined.

***Results:*** Sixty-four patients were male and 31 were female. The average age of male and females was 62.90 ± 11.54 and 61.61 ± 11.98 years, respectively. A significant inverse linear correlation was found between NT-proBNP and FEV1 (P < 0.001, r = -0.367), FVC (P < 0.001, r = -0.444), TLC (P = 0.022, r = -0.238), maximal midexpiratory flow (MMEF) (P = 0.047, r = -0.207) and left ventricular ejection fraction (LVEF) (P < 0.001, r = -0.461). A significant positive linear correlation was found between NT-proBNP and FEV1/FVC (P = 0.013, r = 0.257), RV/TLC (P = 0.003, r=0.303) and 5 Hz Raw (r = 0.231, P = 0.024).

***Conclusion:*** This study showed that, both restrictive and obstructive ventilator impairments can occur in chronic CHF and as NT-proBNP increases appropriate to hemodynamic deterioration, pulmonary dysfunction increases.

## Introduction

Heart failure (HF) is a clinical syndrome which occurs due to hereditary or acquired abnormality of structure or function of the heart. The prevalence of HF increases with age and affects 6%-10% of people older than 65 years.

Any changes in the structure or function of the LV (left ventricle), would predispose the patient to HF. According to the pathophysiology, patients are mainly divided into two groups; systolic and diastolic heart failure.

In industrial societies, coronary artery disease (CAD) is known as the major cause of HF both in men and women (60%-70% of cases). In 75% of the patients suffering from CAD, hypertension is an added risk for development of HF.^1^

Routine preclinical tests for hospitalized patient with HF include routine laboratory tests, electrocardiography, chest radiography (CXR), two-dimensional Doppler echocardiography and biomarkers. These biomarkers are BNP and its precursor which are released from the dysfunctional heart.^1^

BNP is a peptide hormone that is released from the cardiac ventricles as a result of myocyte stretch. This hormone is an inactive pro-hormones that would be broken into two hormones; Active BNP and inactive NT-proBNP.

The pro-hormone is released due to the hemodynamic stress such as ventricular dilatation, ventricular hypertrophy and increased ventricular wall tension. Systemic effects of BNP include vasodilatation, increased urine output with high sodium level, inhibition of the nervous system and the rennin-angiotensin-aldosterone system.^2,3^

Prognostic significance of BNP and NT-pro BNP has been widely studied in patients with heart failure, and the levels of pro-hormones are associated with hemodynamic status in patients with HF and are strong predictor of morbidity and mortality.^2^

Restrictive pattern, characterized by decreased vital capacity (VC) and total lung capacity (TLC) and impaired gas exchange have been recognized in the patients with CHF. On the other hands, small airway obstruction and the airway hyper-responsiveness are reported in patients with CHF.^4-7^ There are several mechanisms such as interstitial edema and airway mucosal vasodilatation, and also vagotonia which result in decreased airway cross-section and airway hyper-responsiveness in CHF.^8,9^ Inhaled glucocorticoids can reduce the airway obstruction, so the inflammatory mediators such as arachidonic acid metabolites, platelet-activating factor, TNF, IL1 and IL6 may play a role in bronchial hyper-responsiveness. Glucocorticoids act in several ways such as inhibition of histamine and IgE dependent LTC4 release and reduction of vascular permeability and mucus secretion.^9^ Increased vagal efferent activity resulting from activation of sensory-neural terminals in the lower airways following edema and hyperemia may lead to bronchospasm. The improvement of forced expiratory volume in one second (FEV1), maximal midexpiratory flow (MMEF) and maximum voluntary ventilation (MVV) following the inhalation of ipratropium bromide confirms the involvement of vagal mechanisms in bronchial obstruction in the patients with CHF. Possible involvement of angiotensin converting enzyme inhibitors in bronchial hyper-responsiveness in patients under treatment for CHF has been studied.^10^

Since the respiratory dysfunction is definitely observed in patients with heart failure and NT-proBNP level is associated with the severity of cardiac dysfunction, the association between NT-proBNP and lung function is the subject of this study.

The aim of this study is to evaluate the relationship between the NT-proBNP levels and the severity of lung function impairment in the patients with chronic CHF.

## Materials and methods

In a cross-sectional analytic study conducted at the Tuberculosis and Lung Disease Research Center at Tabriz University of Medical Sciences. The relationship between the NT-proBNP levels and the severity of lung function impairment studied in patients with CHF.

Patients with a diagnosis of chronic systolic HF were enrolled in this study. After a thorough physical examination, CXR and echocardiography were performed. All patients with known lung diseases, respiratory infections and who have a history of smoking were excluded from study.

The included patients were referred to the pulmonary function laboratory and the lung function tests such as spirometry, body plethysmography and impulse oscillometry (IOS) were performed. Then two ml of venous blood sample were taken from the patients and the plasma level of NT-pro BNP was measured by electrochemiluminesence.

The studied variables were age, sex, severity of congestive heart failure, EF, plasma levels of NT-pro BNP, FEV1, forced vital capacity (FVC), FEV1/FVC, TLC, residual volume (RV), RV/TLC, intrathoracic gas volume (ITGV), ITGV/TLC, airway resistance at 5 and 20 Hz (Raw at 5 Hz, Raw at 20 Hz ) and MMEF rate.

### Statistical analysis

Estimated sample size with α= 0.05 and power= 0.80 and by using power and sample size calculation software version 2.1.3 was 87 patients and we enrolled 95 cases. We used simple random sampling method. Statistical analysis performed by SPSS version 6. For quantitative data analysis, analysis of variance (ANOVA) test and for qualitative data analysis, χ^2^ test were used and then P<0.05 considered significant.

## Results

A total of 95 patients; (64 male and 31 female) with CHF were enrolled. The average age of males and females was 62.90 ± 11.54 and 61.61 ± 11.98 years, respectively.

The main cause of heart failure was CAD in 69 cases (72.6%), and other causes were hypertension 14 (14.7%) cases, cardiomyopathy 6 (6.3%) cases, valvular diseases 4 (4.2%) cases and myocarditis 2 (2.1%) cases.

The average LVEF in males and females was 30.26 ± 9.25 and 29.35 ± 7.22%, respectively.

The average NT-proBNP in males and females was 1358.40 ± 2649.03 and 465.06 ± 624.42, respectively (P=0.013).

There was an significant inverse linear correlation between NT-proBNP and FEV1 (P<0.001, r = -0.367), FVC (P<0.001, r = -0.444), TLC (P=0.022, r = -0.238), MMEF (P =0.047, r = -0.207) and LVEF (P<0.001, r = -0.461).

On the other hand, there was a significant linear correlation between NT-proBNP and FEV1/FVC (P=0.013, r = 0.257), RV/TLC (P = 0.003, r=0.303) and 5 Hz Raw (r = 0.231, P= 0.024).

**Figure 1:**
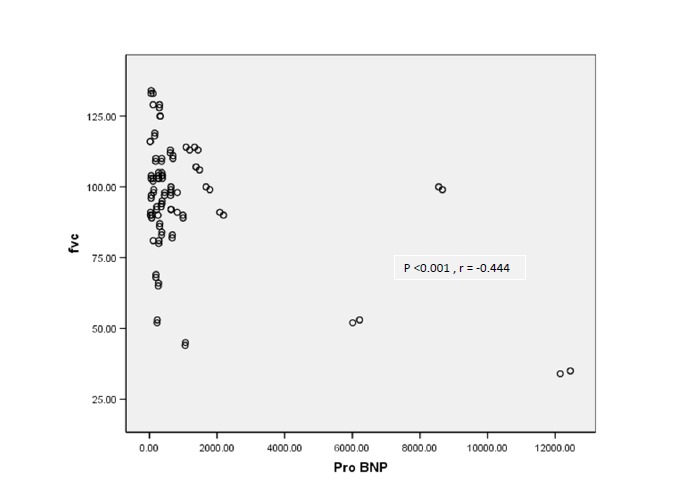
Relation between NT-pro BNP and forced vital capacity

**Figure 2:**
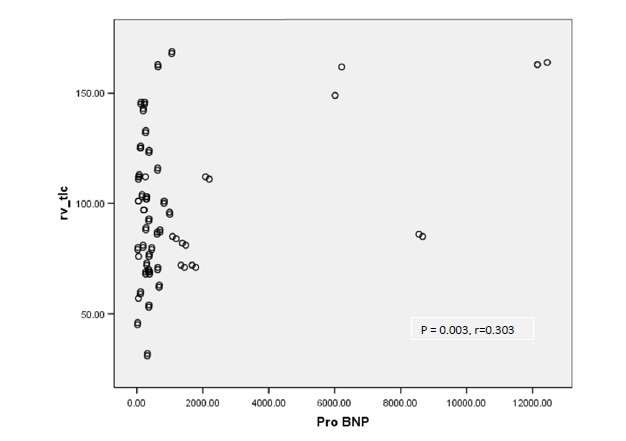
Relation between NT-pro BNP and residual volume over total lung capacity.

There was not any significant linear correlation between NT-proBNP and RV (P= 0.563, r = 0.061), ITGV (P =0.235, r = 0.125), ITGV/TLC (P = 0.201, r = 0.135) and Raw 20 Htz (P = 0.988, r=0.002). Relation between NT-proBNP and FVC and RV/TLC are illustrated in Figures 1 and 2.

## Discussion

CHF is a common disease that its prevalence is increasing in industrial countries.^11-13^

Pulmonary manifestations of heart failure are very well known. In the previous studies, restrictive and obstructive ventilatory impairments and airway hyper reactivity have been shown.^4-7^

In spite of numerous studies, the main mechanism of lung dysfunctions is unknown, but various mechanisms, including; interstitial edema, airways mucosal edema, and vasodilation of bronchiolar vessels have been proposed. These changes lead to a reduction in the cross section of airways and vagotonia.^8,9^ Moreover, stimulation of sensory-neural endings in lower airways by edema and hyperemia-induced efferent vagal activity may play a role in bronchoconstriction.^9,10^

Measurement of NT-proBNP is a simple, noninvasive and available tool to put the possibility of heart failure and can assist to diagnose HF from other causes of acute dyspnea in patients presenting to the emergency room.^14-23^ This marker is a cardiac neurohormone that is released by ventricles in response to overload and increase in volume of ventricles.^17^

There are some studies performed on the serum level of NT-proBNP in pulmonary diseases. For example, a study was carried out by Maeder et al.^24^ They found that the levels of NT-proBNP and BNP in patients with pulmonary diseases are significantly related to the levels of VO2 max and FEV1.^24^

Lee et al.^25^ measured the level of NT-proBNP in patients with Chronic Obstructive Pulmonary Disease. The NT-proBNP levels were increased and associated with mortality of these patients.

Wange et al.^26^ carried out a study in Detroit (USA) to examine the level of NT-proBNP in critically ill patients. There was a meaningful association between the level of NT-proBNP and respiratory failure among patients.^26^

In our study, the level of NT-proBNP was measured in patients with heart failure. There was a significant inverse linear relationship between serum levels of NT-proBNP and left ventricular ejection fraction (P>0.001 and r=-0.461). This is in agreement with findings of previous studies which showed inverse relationship between serum levels of NT-proBNP severity of left ventricular dysfunction. On the other hand there was a direct significant linear relationship between left ventricular ejection fraction (LVEF) and FEV1, FVC and MMEF. We also found a significant inverse linear relationship between EF and Raw 5 Hz. These findings are consistent with the results of previous research which showed obstructive and restrictive ventilatory impairments in patients with heart failure.^4-7^ These findings suggest that pulmonary dysfunction is related to the severity of heart failure.

We showed a significant inverse linear relationship between NT-proBNP levels and FVC, FEV1, TLC and MMEF. Also, there was a significant linear relationship between NT-proBNP levels and FEV1/FVC, RV/TLC, and Raw 5 Hz. Maeder et al, reported similar association between the serum NT-proBNP and FEV1.^24^ There was no significant association was found between NT-proBNP and Raw 20 Hz in our study.

The above results suggest that with an increase in NT-proBNP level, pulmonary volume decreases significantly. Since the level of the aforementioned neurohormone is directly influenced by cardiac output^2,3^, it can be concluded that with an increase in the severity of hemodynamic disturbance, lung volume decreases as a result of congestion. On the other hand, reduction in MMEF and increase in RV/TLC associated with NT-proBNP level elevation, are in favor of obstructive ventilator impairment in these patients.

The main innovation of this study is direct measurement of airway resistance by IOS which showed that small airway resistance increases with intensification of hemodynamic disturbance reflected by elevated NT-proBNP level. This increase in airway resistance explains the decrease in MMEF and the increase in RV/TLC.

## Conclusion

The main mechanism of obstructive changes in lung function in CHF has not been clarified, but some mechanisms including; airway mucosal edema, stimulation of nerve endings, vagotonia, or activation of inflammatory processes has been suggested. However, considering the evident relationship between pulmonary dysfunction and hemodynamic changes, it is likely that other mechanisms involved in pulmonary dysfunction may contribute to the development of hyperemia and congestion.

## Acknowledgments

We thank Tabriz Danesh Pathobiology Laboratory directorship for their kindly contribution in measurement of NT-proBNP on our samples. We also acknowledge Mrs.Mozhgan Khosravi for her contribution in this study by performing pulmonary function tests at Shahid Madani pulmonary function laboratory.

## Ethical issues

The research purposes were explained to the patients and informed written consent was obtained. The patients were assured about their personal information secrecy. According to the research design of study, no costs were paid by the patients for performed tests.

## Competing interests

Authors declare no conflict of interests in this study.
